# The Value of Pressure in Semi-Malignant Mammary Tumours

**Published:** 1876-09

**Authors:** 


The Value of Pressure in Semimalignant Mammary Tumours.—The suggestion contained in the following extract from a clinical lecture, by Doctor George Buchanan, Professor of

Clinical Surgery in the Univesity of Glasgow, is so valuable that we give it prominence :—

There is a kind of tumour which belongs to the simple, or non-malignant fibrous kind, which partakes of malignancy, inasmuch as it returns after removal. Such is the tumour which used to be called recurring fibrous tumour. The question I am going to discuss is not so much the possibility of treating these tumours medically in the way of palliation, but particularly with regard to surgical removal. I wish, however, to tell you at the outset that, because a person has a well-defined tumour in the mamma, it is not absolutely necessary to excise it. I shall say nothing at present with regard to removal by caustics; but it has fallen within my own experience to have seen several most remarkable examples of the disappearance, I might almost use the term cure, of tumour by pressure; and that information is, I think, of great value, because in many cases, from the constitution, or the age of the patient, or from the implication of the neighboring parts, you could not, with any degree of conscientiousness, recommend removal of the tumour; but I could show you ladies in Glasgow, at the present day, who have had tumours in their mammæ, and who are now absolutely free fron the disease by the application of careful and well-directed pressure. You are aware that pressure will cause absorption, both of normal and of abnormal tissues; and you are probably aware that, if a person have an aneurism of the aorta, and if the aneurism continues to grow, it not unfrequently happens, through the tumour pressing upon the sternum, that it gradually induces absorption of the bone until it appears underneath the skin, and if not arrested it spontaneously bursts, and causes loss of life. We are all aware of the importance of pressure in assisting the absorption of abnormal fluids; as by the use of a splint and bandage in case of effusion into joints. In the same way, pressure, well directed to the breast, has a remarkable effect in causing the absorption of tumours; and I now am in the habit of ordering the application of a properly-prepared apparatus in cases where, either from the situation, or the implication of the neighboring parts, I consider that the operation of excising the mamma would be unadvisble.—*Med. and Surg. Reporter.*

